# Oxidative Stress Is a Mediator for Increased Lipid Accumulation in a Newly Isolated *Dunaliella salina* Strain

**DOI:** 10.1371/journal.pone.0091957

**Published:** 2014-03-20

**Authors:** Kaan Yilancioglu, Murat Cokol, Inanc Pastirmaci, Batu Erman, Selim Cetiner

**Affiliations:** 1 Faculty of Engineering and Natural Sciences, Sabanci University, Orhanlı, Istanbul, Turkey; 2 Sabanci University Nanotechnology Research and Application Center, Orhanlı, Istanbul, Turkey; Mount Allison University, Canada

## Abstract

Green algae offer sustainable, clean and eco-friendly energy resource. However, production efficiency needs to be improved. Increasing cellular lipid levels by nitrogen depletion is one of the most studied strategies. Despite this, the underlying physiological and biochemical mechanisms of this response have not been well defined. Algae species adapted to hypersaline conditions can be cultivated in salty waters which are not useful for agriculture or consumption. Due to their inherent extreme cultivation conditions, use of hypersaline algae species is better suited for avoiding culture contamination issues. In this study, we identified a new halophilic *Dunaliella salina* strain by using 18S ribosomal RNA gene sequencing. We found that growth and biomass productivities of this strain were directly related to nitrogen levels, as the highest biomass concentration under 0.05 mM or 5 mM nitrogen regimes were 495 mg/l and 1409 mg/l, respectively. We also confirmed that nitrogen limitation increased cellular lipid content up to 35% under 0.05 mM nitrogen concentration. In order to gain insight into the mechanisms of this phenomenon, we applied fluorometric, flow cytometric and spectrophotometric methods to measure oxidative stress and enzymatic defence mechanisms. Under nitrogen depleted cultivation conditions, we observed increased lipid peroxidation by measuring an important oxidative stress marker, malondialdehyde and enhanced activation of catalase, ascorbate peroxidase and superoxide dismutase antioxidant enzymes. These observations indicated that oxidative stress is accompanied by increased lipid content in the green alga. In addition, we also showed that at optimum cultivation conditions, inducing oxidative stress by application of exogenous H_2_O_2_ leads to increased cellular lipid content up to 44% when compared with non-treated control groups. Our results support that oxidative stress and lipid overproduction are linked. Importantly, these results also suggest that oxidative stress mediates lipid accumulation. Understanding such relationships may provide guidance for efficient production of algal biodiesels.

## Introduction

The idea of using biofuels has gained prominence, since they provide a cleaner alternative to the currently used fossil fuels. It has recently been estimated that utilization of biofuels will result in a 30% decrease in CO_2_ emissions in the United States. Biofuels can be derived from different kinds of resources including microalgae, animal fats, soybeans, corns and other oil crops. While none of these options currently has the efficiency to produce the required amounts of biofuel [1], microalgae are considered the most promising venue of biofuel production due to their ease of cultivation, sustainability, and compliance in altering their lipid content resulting in higher biofuel production.

High lipid accumulation and biomass productivity are the two manifestly desired phenotypes in algae for biodiesel production. However, various studies conducted under nutrient depleted conditions have demonstrated that biomass productivity and lipid accumulation are negatively related [2]. These studies have established that stress conditions, which by definition reduce the biomass production, increase lipid content of algae. This problem was addressed by using a two-stage reactor where algal species such as *Oocysti sp.* and *amphora sp.* are grown in optimal conditions for maximum biomass, followed by stress conditions for maximum lipid accumulation [3]. Within this context, nitrogen depletion can be still considered as a strategy for increasing lipid accumulation since it has been still defined as one of the best lipid accumulator stress condition in algae to date. However the mechanistic insights of this phenomenon are still needed.

Nitrogen deprivation as a stress condition is known to maximize the lipid content up to 90% [4]. However, underlying mechanisms have not been well described in terms of its physiological and molecular aspects. Despite the fact that oxygen itself is not harmful for cells, the presence of reactive oxygen species (ROS) may lead to oxidative damage to the cellular environment, ultimately leading to toxicity resulting from excessive reactive oxygen stress [5]. Redox reactions of the reactive forms of oxygen including hydrogen peroxide (H_2_O_2_), superoxide (O_2_^−^) or hydroxyl (OH^−^) radicals with cellular lipids, proteins, and DNA result in oxidative stress [6]. A previous study showed that nitrogen depletion results in the co-occurrence of ROS species and lipid accumulation in diatoms [7]. Association of increased reactive oxygen species levels and cellular lipid accumulation under different environmental stress conditions was also shown in green microalgae [8] ROS is known to be an important factor in cellular response and it is well established that ROS increases when microalgae are exposed to various stresses. However, a mechanistic understanding of the connection between ROS increase and increased lipid accumulation in algae species requires further investigation [9].

Nitrogen depleted conditions trigger reactive oxygen species accumulation, increased cellular lipid content and protein production impairment. However, the temporal order and the causal links between these events are yet to be explored. Here, we aimed at finding the relationship between oxidative stress and increased cellular lipid content under nitrogen depleted conditions in a hypersaline green alga in order to have a better understanding of this phenomenon.

*Dunaliella* genus [10] is one of the microalgae genus that has been considered for lipid production. *Dunaliella* species are particularly attractive due to their strong resistance characteristics to various unfavourable environmental conditions such as high salinity. Obtaining such strong algal species for lipid production under conditions that are otherwise not useful is an important economical consideration in terms of biodiesel production. In addition, cultivation of algae species in freshwaters may not be feasible due to the limited supply and population expansion. Understanding the mechanisms behind increased lipid accumulation in halophilic *Dunaliella* in response to different stress conditions, especially nitrogen depletion, is crucial to enable key manipulations at the genetic, biochemical and physiological level for decreasing biodiesel production costs.

## Materials and Methods

### Organism and Culture Conditions

*Dunaliella tertiolecta* (*D.t.* #LB999) and *Chlamydomonas reinhardtii* (*C.r.* #90) were obtained from UTEX, Collection Culture of Algae, USA and cultivated artificial sea water medium and soil extract medium as instructed by the culture collection protocols respectively. The alga used in this study was isolated from the hypersaline lake “Tuz”, which is located in Middle Anatolia, Turkey with the research permission of Republic of Turkey Ministry of Food, Agriculture and Livestock (Permit issue: B.12.0.TAG.404.03.10.03.03–1607). Field studies did not involve endangered or protected species. Collection of water samples was done and isolation location was recorded using a GPS device as 39°4′23.97″K - 33°24′33.11″E at the southern east part of Sereflikochisar province. 10 ml water samples were collected and enriched with the same volume of Bold’s Basal Medium (BBM) modified by addition of 5% NaCl. BBM was pH 7.4 and consisted of 5 mM NaNO_3_ along with CaCl_2_·2H_2_O 0.17 mM, MgSO_4_·7H_2_O 0.3 mM, K_2_HPO_4_ 0.43 mM, KH_2_PO_4_ 1.29 mM, Na_2_EDTA·2H_2_O 2 mM, FeCl_3_·6H_2_O 0.36 mM, MnCl_2_ **·**4H_2_O 0.21 mM, ZnCl_2_ 0.037 mM, CoCl_2_ **·**6H_2_O 0.0084 mM, Na_2_MoO_4_ **·**2H_2_O 0.017 mM, Vitamin B_12_ 0.1 mM and 5 mM NaCO_3_ was supplied as carbon source.

Water samples were plated on petri dishes with modified BBM 5% NaCl and 1% bacteriological agar, while same water samples were also subjected to dilutions with fresh modified BBM 5% NaCl in 48-well plates (1∶2, 1∶4, 1∶8, 1∶16, 1∶32, 1∶64) to obtain monocultures. After 2 and 4 weeks cultivation periods of 48-well plate liquid cultures and petri dishes, respectively, clones were isolated. These clones were transferred to fresh mediums in 100 ml canonical flasks in a final volume of 25 ml for obtaining cell stocks at 25°C under continuous shaking and photon irradiance of 80 rpm and 150 μEm^−2^s^−1^.

All experiments were carried out under same cultivation conditions in 250 ml batch cultures but different nitrogen concentrations, 0.05, 0.5 and 5 mM NaNO_3_ were used for low, medium and high nitrogen concentrations, respectively. Concentrations ranging between 200 uM and 8 mM were used for H_2_O_2_ experiments.

### Isolation and Purification of DNA and Amplification of 18S rRNA Encoding Gene

DNA isolation was done by using DNeasy Plant Mini Kit (Qiagen) as instructed by the manufacturer. Quantification of the genomic DNA obtained and assessment of its purity was done on a Nanodrop Spectrophotometer ND-1000 (Thermo Scientific) and on 1% agarose gel elecrophoresis. MA1 [5′-CGGGATCCGTAGTCATATGCTTGTCTC-3′] and MA2 [5-GGAATTCCTTCTGCAGGTTCACC-3′] were designed from 18S rDNA genes and were previously reported by Olmos et al [11]. PCR reactions were carried out in a total volume of 50 μl containing 50 ng of chromosomal DNA in dH_2_O and 200 ng MA1 and MA2 conserved primers. The amplification was carried out using 30 cycles in a MJ Mini Personal Thermal Cycler (BioRad), with a T_m_ of 52°C for all reactions. One cycle consisted of 1 minute at 95°C, 1 minute at 52°C and 2 minutes at 72°C.

### Sequencing and Phylogenetic Analysis

MA1–MA2 PCR products were utilized to carry out sequencing reactions after purification with a QIAquick PCR purification kit (Qiagen). The sequencing reactions were run by MCLAB (San Francisco, CA), employing primers MA1–MA2 in both reverse and forward directions. DNA sequences were imported to BLAST for identification and to search for phylogenetic relationship correlations between other 18S rDNA gene sequences of *Dunaliella* species/strains deposited in NCBI Gene Bank. Dendrogram data generated by BLAST was converted into newick format and submitted to Phyfi [12] for generating phylogenetic tree.

### Growth Analysis

Specific growth rate and biomass productivity was calculated according to the equation; 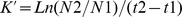 where 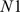 and 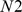, biomass at time1 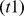 and time2 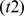 respectively 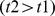 [13]. Divisions per day and the generation (doubling) time were calculated according to the equations below:
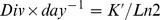


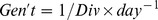



### Extraction and Measurements of Lipid Contents and Fluorescence Microscopy

Lipid was extracted according to Bligh and Dyer wet extraction method. Briefly, to a 15 ml glass vial containing 100 mg dried algal biomass, 2 ml methanol and 1 ml chloroform were added and kept for 24 h at 25°C. The mixture was then vortexed and sonicated for 10 minutes. One milliliter of chloroform was again added, and the mixture shaken vigorously for 1 min. Subsequently, 1.8 ml of distilled water was added and the mixture vortexed again for 2 min. The aqueous and organic phases were separated by centrifugation for 15 min at 2,000 rpm. The lower (organic) phase was transferred into a previously weighed clean vial (V1). Evaporation occurred in a thermo-block at 95°C, and the residue was further dried at 104°C for 30 min. The weight of the vial was again recorded (V2). Lipid content was calculated by subtracting V1 from V2, and expressed as dcw %. The correspondence between Nile Red fluorescence intensity and % lipid content was determined by plotting relative fluorescence units against % cellular lipid content obtained from triplicate samples. For fluorescence microscopy analysis of Nile Red, cells were stained with 5 μl 0.5 mg/mL Nile Red (Sigma, USA) stock solution after fixing cells with 5% paraformaldehyde and imaged by epi-fluorescence microscopy with a Leica DMR microscope (Leica Microsystems).

### Spectrofluorometric Microplate Analysis for Determination of Lipid and Reactive Oxygen Species Accumulation

Nile red, 9-diethylamino-5H-benzo[alpha]phenoxazine-5-one was first reported that is an excellent vital stain for the detection of intracellular lipid droplets by fluorescence microscopy and flow cytoflourometry [14] and it has been widely used for measuring and comparing cellular lipid content in various organisms in numerous studies [15]–[19]. A stock solution of Nile Red (NR) (Sigma, 72485) was prepared by adding 5 mg of NR to 10 ml of acetone. The solution was kept in a dark colored bottle and stored in the dark at −20°C. 1 ml of algal cells from a culture of 250 ml glass erlenmeyer flasks containing 100 ml growth media with different nitrogen concentrations were transferred to 1.5 ml eppendorf tubes for 5 min centrifugation at 5,000 rpm, washed twice with fresh medium, and measured in a spectrophotometer at 600 nm. Each sample was adjusted to an OD_600_ of 0.3 in a 1 ml final volume by dilution with fresh medium. 5 μl of Nile Red solution was added to each tube and mixed well, followed by 20 min incubation in the dark. Finally, cellular neutral lipids were quantified using a 96-well microplate spectrofluorometry (SpectraMAX GEMINI XS) with an excitation wavelength of 485 nm and an emission wavelength of 612 nm.

Dichloro-dihydro-fluorescein diacetate (DCFH-DA) is the most widely used fluorometric probe for detecting intracellular oxidative stress. This probe is cell-permeable and is hydrolyzed intracellularly to the DCFH carboxylate anion. Two-electron oxidation of DCFH results in the formation of a dichlorofluorescein (DCF) as a fluorescent product. The amount of this fluorescent product is highly correlated with the cellular oxidation/oxidative stress level. Investigators have routinely used DCFH-DA to measure intracellular generation of reactive oxidants in cells in response to intra- or extracellular activation with oxidative stimulus [20]. Determination of ROS production related to oxidative stress was done by using DCFH-DA (Sigma, USA). Vital staining for determination of cell survival under H_2_O_2_ treatment was done by using fluorescein diacetate (FDA) (Sigma, USA). FDA is incorporated into live cells and it is converted into fluorescein by cellular hydrolysis [21]. 0.5 mg/ml stock solutions for both stains were prepared in acetone and the same protocol was used for FDA (Sigma, USA) and DCFH-DA (Sigma, USA) staining as described above for Nile Red staining. Data were recorded as relative fluorescence units [22] for all spectrofluorometric staining experiments.

### Flow Cytometric Analysis for Determination of Lipid and Reactive Oxygen Species Accumulation

5 μl of Nile Red (Sigma, USA) from stock solution (0.5 mg/mL) was added to 1 ml of a cell suspension at an OD_600_ of 0.3 after washing cells twice with fresh medium. This mixture was gently vortexed and incubated for 20 minutes at room temperature in dark. Nile Red uptake was determined using a BD-FACS Canto flow cytometer (Becton Dickinson Instruments) equipped with a 488 nm argon laser. Upon excitation by a 488 nm argon laser, NR exhibits intense yellow-gold fluorescence when dissolved in neutral lipids. The optical system used in the FACS Canto collects yellow and orange light (560–640 nm, corresponding to neutral lipids). Approximately 10,000 cells were analysed using a log amplification of the fluorescent signal. Non-stained cells were used as an autofluorescence control. Nile Red fluorescence was measured using a 488 nm laser and a 556 LP+585/42 band pass filter set on a FACS Canto Flow Cytometer. Data were recorded as mean fluorescence intensity (MFI).

DCFH-DA (Sigma, USA) from stock solution (0.5 mg/ml) was used as described above for flow-cytometric microplate Nile Red staining method in order to analyze cellular oxidative stress status. Briefly, 1 ml of algal cells in different nitrogen concentrations were transferred into 1.5 ml eppendorf tubes, centrifuged 5 min at 5,000 rpm, washed twice with fresh medium, and measured in a spectrophotometer at 600 nm. Each sample was adjusted to an OD_600_ of 0.3 in a 1 ml final volume by dilution with fresh medium. 5 μl of dye solution was added to each tube and mixed well, followed by 20 min incubation in the dark and analyzed. Nonfluorescent DCFH-DA is taken up and converted into diclorodihydrofluorescein (DCF) by the action of cellular esterases. Fluorescence from oxidized DCF was measured using a 488 nm laser and a 556/Long Pass filter set on a BD-FACS Canto Flow Cytometer. Data were expressed as mean fluorescence intensity (MFI). The data of both staining methods were evaluated using Flowjo Ver. 7.6.1 (Tree Star, Inc.).

### Protein, Chlorophyll, Carotenoid, TBARS Analyses and Enzymatic Assays

Total protein isolation was done according to Barbarino et al. [23]. Protein content was determined following the method described by Bradford [24]. Cellular chlorophyll and carotenoid isolations were done using the methanol extraction method. Calculation of chlorophyll and carotenoid contents were carried out using the formula for methanol extraction described by Wellburn [25]. Lipid peroxidation analysis upon different nitrogen concentration regimes was analysed by using thiobarbituric acid reactive substances (TBARS) method described by Sabatini et al [26]. For superoxide dismutase (SOD) analysis 50 mg biomass was homogenized in 2 ml 0.5 M phosphate buffer (pH 7.5) and centrifuged at 13,000 rpm for 10 min at 4°C. SOD activity was determined in the supernatant by inhibition of nitroblue tetrazolium (NBT) using a reaction mixture of 1.5 ml Na_2_CO_3_ (1 M), 200 μl methionine (200 mM), 100 μl NBT (2.25 mM), 100 μl EDTA (3 mM), 100 μl riboflavin (60 ?M) and 1.5 ml phosphate buffer (pH 7.8, 0.1 M). The absorbance was recorded at 560 nm. One unit of SOD per gram of protein was defined as the amount causing 50% inhibition of photochemical reduction of NBT [27]. For catalase (CAT) analysis, 50 mg biomass was homogenized in 2 ml phosphate buffer (0.5 M, pH 7.5), centrifuged at 12,000 rpm at 4°C for 30 min and supernatant was taken for CAT activity. A reaction mixture containing 1.6 ml phosphate buffer (pH 7.3), 100 μl EDTA (3 mM), 200 μl H_2_O_2_ (0.3%) and 100 μl supernatant (containing enzyme extract) was taken in a cuvette and CAT activity in supernatant was determined by monitoring the disappearance of H_2_O_2_, by measuring a decrease in absorbance at 240 nm against a blank of same reaction mixture without 0.3% H_2_O_2_ [28]. For analysis of ascorbate peroxidase (APX), 50 mg biomass was homogenized in 2 ml phosphate buffer (0.5 M, pH 7.5) and centrifuged at 12,000 rpm at 4°C for 30 min. The supernatant was taken for APX activity. A reaction mixture containing 1 ml phosphate buffer (pH 7.3), 100 μl EDTA (3 mM), 1 ml ascorbate (5 mM), 200 μl H_2_O_2_ (0.3%) and 100 μl supernatant (containing enzyme extract) was prepared in a cuvette. The reaction was followed for 3 min at a wavelength of 290 nm against a blank of same reaction mixture without 0.3% H_2_O_2_ [29].

## Results and Discussion

### Isolation and Identification of the New *Dunaliella salina* Strain

We isolated a new hypersaline microalga from the biggest salt lake of Turkey located in the Middle Anatolian region in July 2011. The microalga, which is unicellular, biflagellate and tolerant up to 20% NaCl was identified as *Dunaliella salina* based on the morphological characterization prior to molecular identification. We observed that cell color was green under normal conditions and the color changed to red under stress conditions. Morphologically, ovoid, spherical and cylindrical cell shapes were observed. Stigma was not clearly visible or diffuse. Large pyrenoid with distict amylosphere was observed and refractile granules are absent. Cellular size was measured as ∼20 μm. These observed characteristics were consistent with previous morphological characteristics of *Dunaliella salina.*

**Figure 1:**
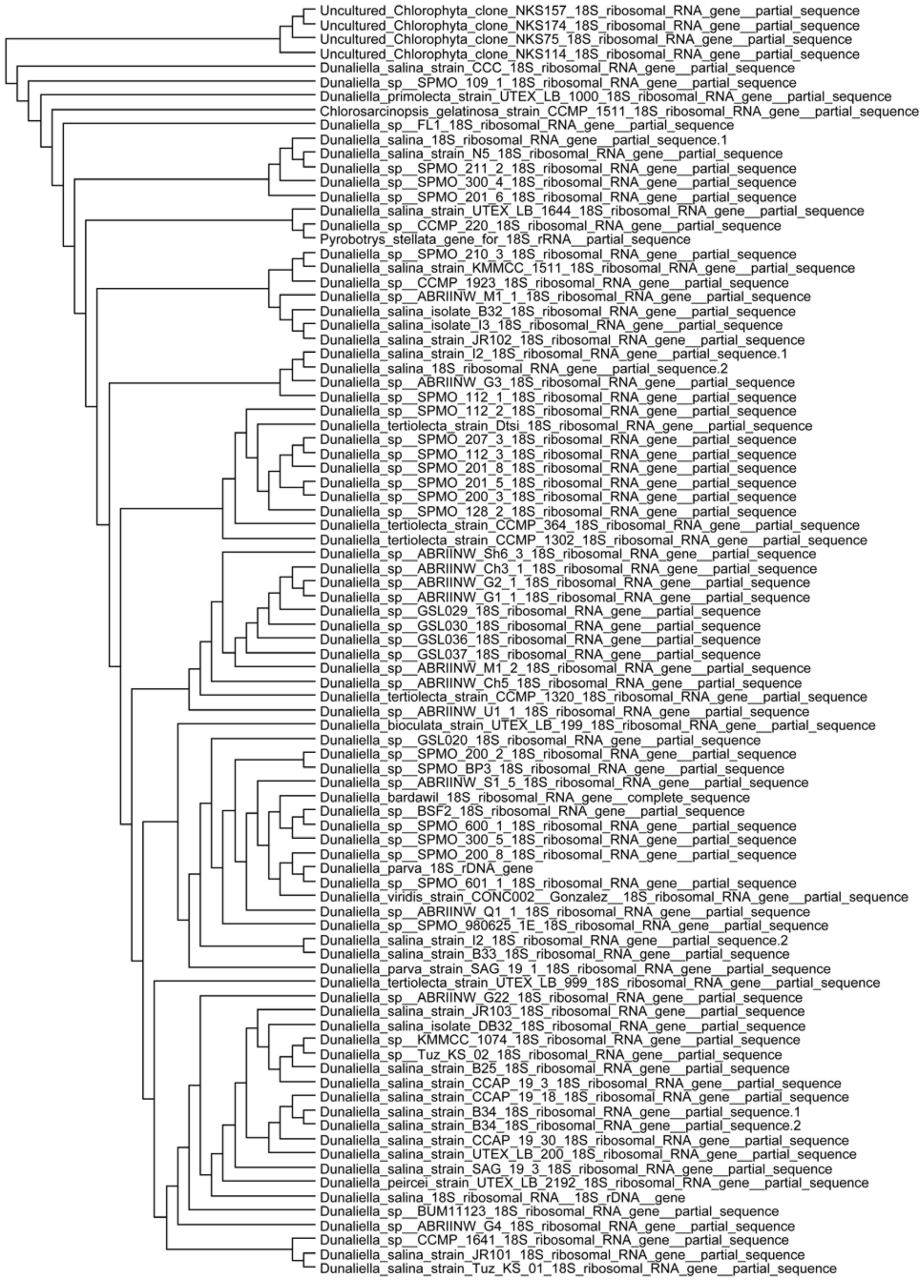
Phylogenetic analysis of *Dunaliella salina strain Tuz_KS_01 (*GeneBank accession no. JX880083). Dendrogram was generated using the neighbor-joining analysis based on 18S rDNA gene sequences. The phylogenetic tree shows the position of *Dunaliella salina strain Tuz_KS_01* (GeneBank accession no. **JX880083**) relative to other species and strains of *Dunaliella* deposited in NCBI GeneBank.

This strain was deposited in −196°C ultra freeze conditions at Sabanci University algae culture collection as *Dunaliella salina strain Tuz_KS_01*. Molecular identification was based on 18S rDNA gene sequence amplification and sequencing. A single 18S rDNA PCR amplicon was about ∼1800 bp in size, in accordance with Olmos et al. [11]. The partial sequence of the 18S rDNA encoding gene was submitted to National Center for Biotechnology Information (NCBI) GeneBank database as *Dunaliella salina strain Tuz_KS_01* (GeneBank accession no. **JX880083**). According to Basic Local Aligment Search Tool (BLAST) analysis, the isolated sequence had very high percentage of identity with other deposited 18S rDNA sequences of *Dunaliella* species shown in the phylogenetic tree plotted in Figure 1. Molecular identification showed a high percentage identity to *Dunaliella salina* strains (Identity 93%, Query Coverage 70%).

### Growth Analysis of the New Dunaliella Strain Under Different Nitrogen Concentrations

**Figure 2:**
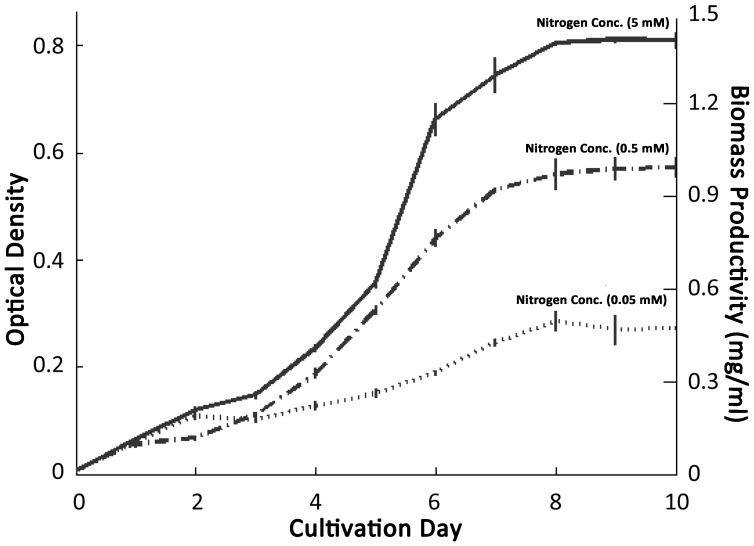
Effects of different nitrogen concentrations on the growth and biomass productivity of *Dunaliella salina strain Tuz_KS_01 (*GeneBank accession no. JX880083). 0.05_3_ are referred as low, medium and high nitrogen concentrations respectively. Shown OD_600_ optical density and biomass values are the means of three replicates. The error bars correspond to ±1 SD of triplicate optical density measurements.

The newly isolated halotolerant *Dunaliella salina* strain was cultivated under different nitrogen concentrations for growth analysis. 0.05 mM, 0.5 mM and 5 mM NaNO_3_ concentrations were considered as low, medium and high nitrogen concentrations. Specific growth rates and/or average biomass productivities (ABP) were found to be 370 mg/dayL, 430 mg/dayL, 520 mg/dayL for low, medium and high nitrogen groups respectively. The highest biomass concentration under low nitrogen regimes was 495 mg/l, compared with 994 mg/l and 1409 mg/l for medium and high nitrogen regimes shown in Figure 2. Other kinetic growth data including doubling time/generation time and division per day were also calculated. Doubling times were calculated as 1.84, 1.59, and 1.32 whereas division of cells per day was calculated as 0.54, 0.62, and 0.75 for low, medium and high nitrogen concentration groups respectively.

Biomass productivity is one of the most important parameters for the feasibility of utilizing algal oil for biodiesel production. Hence, numerous studies have been conducted in various algal species. Tang et al. (2011) demonstrated that *Dunaliella tertiolecta* had a highest biomass concentration of ∼400 mg/l [30]. In another study conducted by Ho et al. (2010) the optimal biomass productivity of freshwater alga *Scenedesmus obliquus* were found to be 292.50 mg/dayL [31]. Griffiths et al. (2012) studied 11 different algal species including freshwater, marine and halotolerant species and demonstrated their biomass productivities [32]. Compared to the reported biomass productivities in previous studies, both the highest biomass concentration and average biomass productivity of isolated *Dunaliella salina strain Tuz_KS_01* showed very good potential, eventhough small batch cultivation process was utilized instead of using more sophisticated and efficient cultivation systems.

### Lipid Accumulation Analysis of the New *Dunaliella salina* Strain Under Different Nitrogen Concentrations

**Figure 3:**
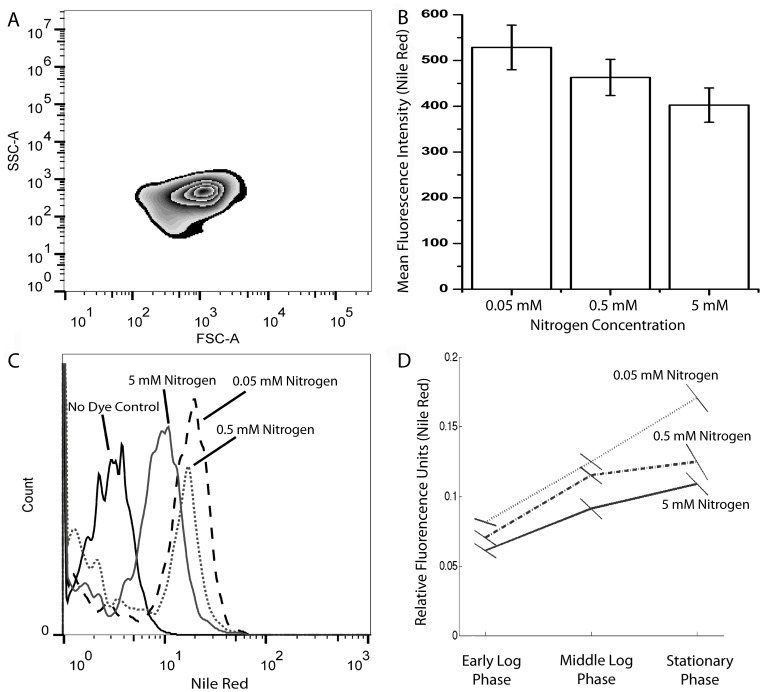
Lipid content analysis of *Dunaliella salina strain Tuz_KS_01* under different nitrogen conditions. **A)** Zebra-plot of SSC (Side Scatter) and FSC (Forward Scatter) expressing cellular granulation and cellular size, respectively, under a representative nitrogen depletion condition (0.05 mM). **B)** A representative histogram of flow-cytometric analysis of lipid contents under different nitrogen concentrations **C)** Mean fluorescent intensities (MFI) of flow cytometric analysis. **D)** Fluorometric microplate Nile Red analysis of early logarithmic, late logarithmic and stationary growth phases. Data represent the mean values of triplicates. Standard error for each triplicate is shown as tilted lines for clarity, where the minimum and maximum y values of each line corresponds to ±1 SE.

Experimental groups were subjected to flow cytometric Nile red analysis for measuring lipid accumulation at single cell level in stationary growth phase. Cell populations were chosen according to cellular size and granulation as quantified by forward and side scatter values of populations, respectively which is demonstrated in Figure 3A. Figure 3B shows a representative flow cytometric analysis of lipid contents under different nitrogen concentrations, indicating high lipid content under low nitrogen condition. Figure 3C shows the mean and standard error for three biological replicates, clearly exhibiting a negative relationship between lipid accumulation and nitrogen levels. Therefore, cultivation under low nitrogen conditions stimulates lipid accumulation in *Dunaliella salina strain Tuz_KS_01*, in agreement with previous studies [33], [34].

In addition, we measured lipid accumulation of the green alga under different nitrogen levels using spectrofluorophotometer [35] at early-logarithmic, late-logarithmic and stationary growth phases. While the cellular lipid accumulation in early and late logarithmic phases did not show a drastic change, we observed a marked increase of lipid content in the stationary phase, which was significantly higher under low nitrogen cultivation conditions shown in Figure 3D.

**Figure 4:**
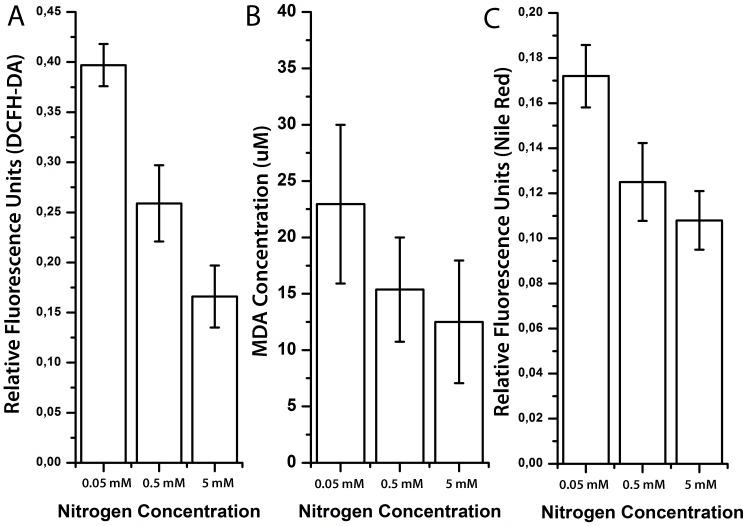
Oxidative stress and lipid accumulation under different nitrogen concentrations. **A)** Fluorometric microplate DCFH-DA analysis under different nitrogen concentrations. **B)** TBARS analysis for lipid peroxidation under different nitrogen concentrations. **C)** Fluorometric microplate Nile-Red analysis under different nitrogen concentrations. Data represent the mean values of triplicates ±1 SE.

Nitrogen depletion is well known to result in increased lipid accumulation in algal species [36], eventhough its association with other related factors has not been well defined. To demonstrate the oxidative stress under nitrogen limited cultivation conditions, fluorometric DCFH-DA and related TBARS lipid peroxidation assays were utilized. According to these results, increased ROS production (Figure 4A) and lipid peroxidation (Figure 4B) were both observed under nitrogen limited conditions, in association with increased lipid accumulation especially at stationary phase shown in Figure 4C.

### Antioxidant Enzyme Activities, Pigment Composition and Protein Analyses Under Different Nitrogen Concentrations

ROS accumulation is prevented by a powerful intrinsic antioxidant system in photosynthetic organisms, involving enzymes such as superoxide dismutase (SOD), catalase (CAT), ascorbate peroxidase (APX) [37]. Next, we analyzed these three oxidative stress indicator antioxidant enzymes to demonstrate the effect of nitrogen depletion on the intracellular oxidative stress status as also supplementary to DCFH-DA measurements.

**Figure 5:**
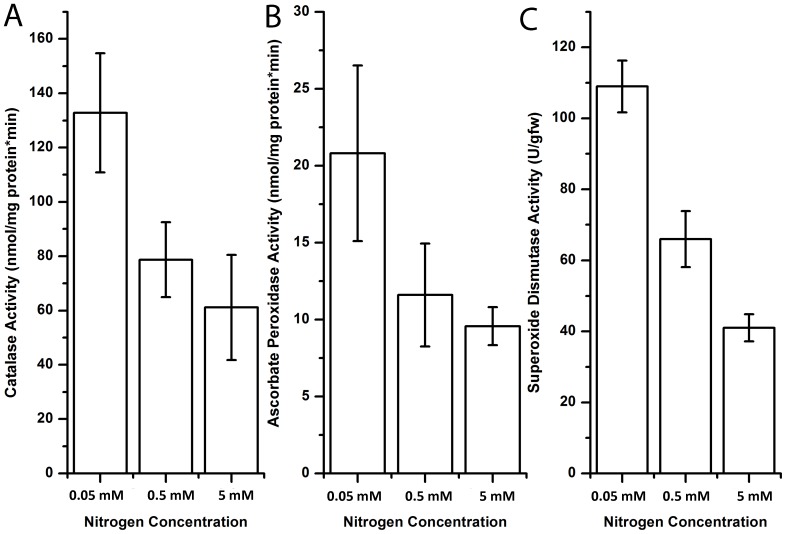
Antioxidant enzyme activities under different nitrogen concentrations. **A–C)** Spectrophotometric enzymatic assays for catalase (CAT), ascorbate peroxidase (APX) and superoxide dismutase (SOD). Data demonstrate the mean values of triplicates ±1 SE.

Intracellular SOD converts O_2_^−^ to O_2_ and H_2_O_2_, acting as the first line of defence against oxidative stress [38]. We observed that SOD activity of cells increased under low nitrogen concentrations, especially at stationary growth phase. This data suggests that superoxides may be elevated under nitrogen depleted conditions, necessitating increased SOD activity (Figure 5A). CAT is a heme-containing enzyme that catalyzes the conversion of H_2_O_2_ into oxygen and water [37]. APX is involved in the ascorbate-glutathione cycle occuring in chloroplasts, cytoplasm, mitochondria and perixisomes [39]. We observed elevated levels of CAT and APX activity under nitrogen depleted conditions (Figure 5B, 5C). These data strongly indicate that oxidative stress is induced under nitrogen depletion in *Dunaliella salina strain Tuz_KS_01.*

**Figure 6:**
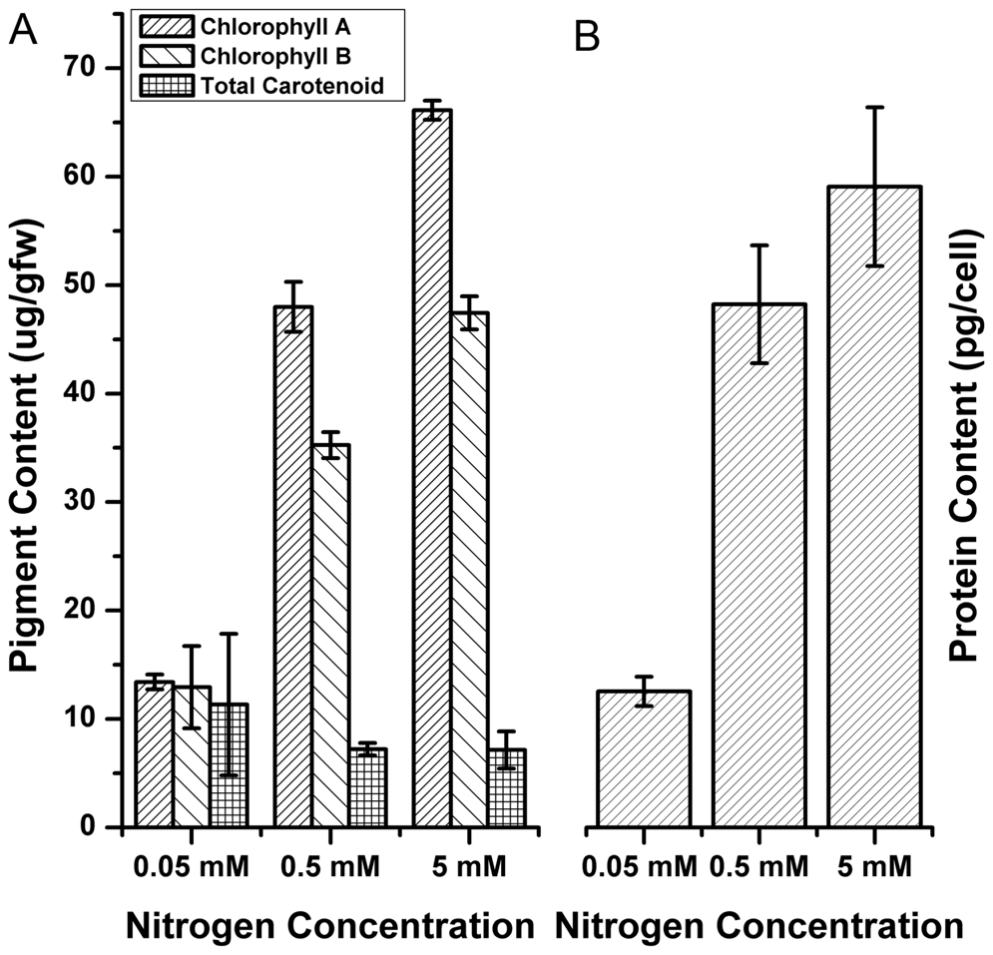
Pigment and protein contents of *Dunaliella salina* strain under different nitrogen concentrations. **A)** Chlorophyll A, B and total carotenoid contents under different nitrogen concentrations. **B)** Protein contents of *Dunaliella salina strain Tuz_KS_01* cells cultivated under different nitrogen concentrations Data demonstrate the mean values of triplicates ±1 SE.

Next, we analyzed the chlorophyll, carotenoid and protein content change in response to nitrogen depletion in *Dunaliella salina strain Tuz_KS_01*. Chlorophyll content is an indicator of photosynthetic efficiency, rate and nitrogen [40] status. Carotenoids can perform an essential role in photoprotection by quenching the triplet chlorophyll and scavenging singlet oxygen and other reactive oxygen species [41]. We observed that chlorophyll A and B levels sharply declined in low nitrogen conditions, as expected (Figure 6A). In contrast, we observed no decrease in total carotenoid contents under nitrogen depletion conditions. This observation suggests that cells increase caroteoid content to overcome with oxidative stress induced by singlet oxygens, similar to SOD activity increase discussed above (Figure 6A).

Upon nitrogen starvation, algal cells reduce the synthesis of protein and nucleic acid synthesis and carbon flow is directed from protein synthesis to fatty acid and carbonhydrate synthesis [42]. We found that neutral lipids increase while the protein content of algal cells was reduced under nitrogen depletion conditions (Figure 6B). This indicates that carbon flow direction is to fatty acid synthesis under nitrogen limitation at stationary phase, in accordance with the literature [43].

### Reactive Oxygen Species (ROS) Production Induces Lipid Accumulation

**Figure 7:**
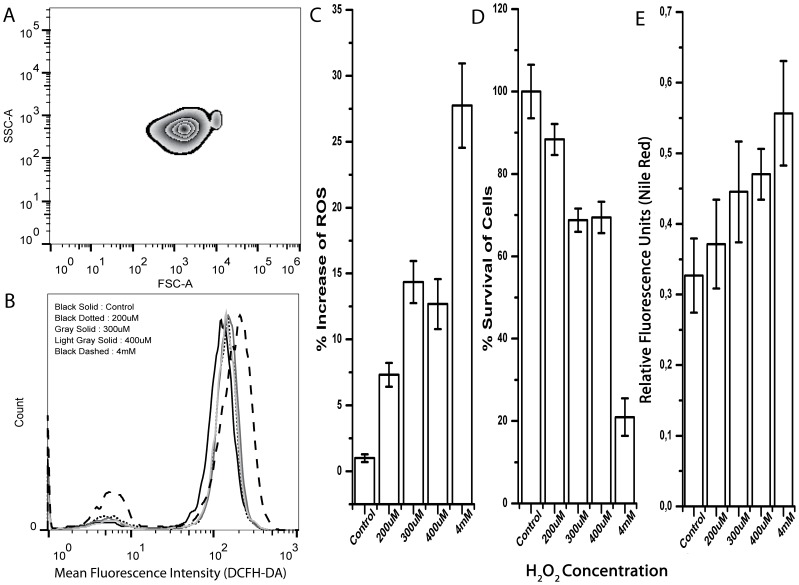
Effect of H_2_O_2_, a known oxidative stress inducer, on *Dunaliella salina strain Tuz_KS_01.* **A)** Zebra-plot of SSC (Side Scatter) and FSC (Forward Scatter) expressing cellular granulation and cellular size under a representative H_2_O_2_ condition (4 mM). **B)** Histogram of flow-cytometric analysis of ROS accumulation under different H_2_O_2_ concentrations. **C)** Percentage increase of ROS production based on flow-cytometric DCFH-DA analysis. **D)** Fluorometric microplate fluorescent diacetate (FDA) survival analysis cultivated under different H_2_O_2_ concentrations. **E)** Fluorometric microplate Nile-Red analysis under different H_2_O_2_ concentrations. Data represent the mean values of triplicates ±1 SE.

Our analysis thus far hints that lipid accumulation might be partially triggered by ROS accumulation and oxidative stress under nitrogen depleted condition (Figure 4). In order to test this hypothesis, we used H_2_O_2_, a well-known oxidative stress inducer [44]. We treated cells at exponential growth phase in high nitrogen containing media (5 mM) with different concentrations of H_2_O_2_ and we measured ROS, via using flowcytometric DCFH-DA method, a robust fluorescent assay [45]. In addition, cell viability was measured by using FDA fluorescent assay while using Nile red staining method for determination of lipid contents under different H_2_O_2_ concentrations. Although cellular size and granularity under H_2_O_2_ treated conditions were similar to previous experimental conditions (Figure 7A and 3A), cell survival under higher H_2_O_2_ concentrations were observed significantly reduced due to the oxidative stress induction (Figure 7B–7C). Although reduction of cell survival is to ∼20% at 4 mM H_2_O_2_ concentration, at this high concentration of H_2_O_2_, new isolate *Dunaliella salina strain Tuz_KS_01* showed highly tolerant characteristics to oxidative stress (Figure 7D).

Lipid accumulation of algae is known to increase by various factors including temperature, excessive light, and pH, which may also related to oxidative stress induced by ROS accumulation [33], [46]. We used fluorometric Nile red analysis for analysis of lipid accumulation under different H_2_O_2_ treatments. We observed increased lipid accumulation with increasing concentrations of H_2_O_2_ (Figure 7E). This result supports that the lipid accumulation observed under nitrogen depletion conditions is at least partly mediated by oxidative stress. Importantly, this result also suggests a method for obtaining high lipid from green alga, namely by growing cells in high nitrogen (optimal) conditions followed by oxidative stress induction at stationary phase. Such a method may be economically more feasible than two stage reactors described above.

**Figure 8:**
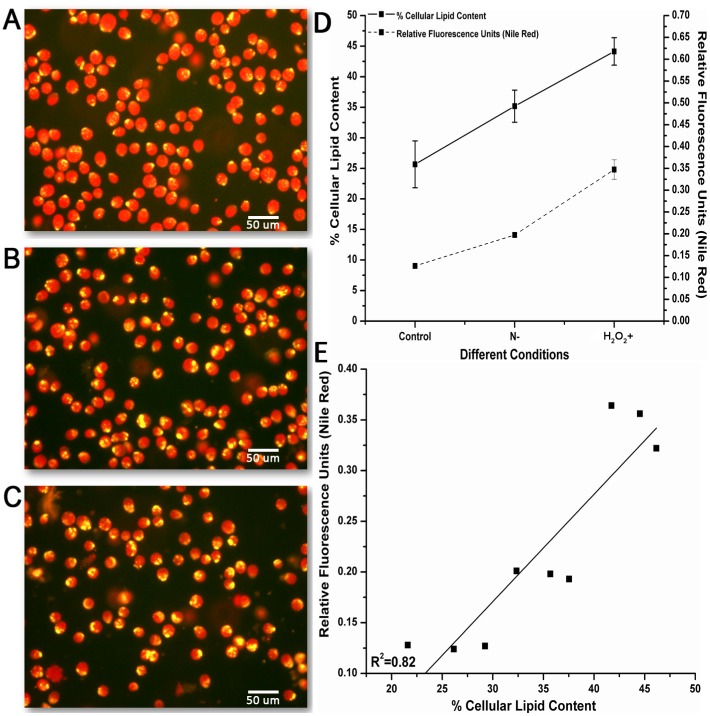
Fluorescence microphotographs of *Dunaliella salina strain Tuz_KS_01* stained with Nile-Red fluorescence dye and screened under 400X magnification. **A)** Control group, 5 mM nitrogen concentration cultivation condition. **B)** Nitrogen limitation group, 0.05 mM nitrogen condentration cultivation condition **C)** Oxidative stress group, 4 mM H_2_O_2_ cultivation condition. Gravimetric lipid content analysis and Nile-Red fluorescence measurement correlation **D)** Gravimetric and fluorometric Nile-Red lipid content analysis of *Dunaliella salina strain Tuz_KS_01* under 0.05 mM nitrogen and 4 mM H_2_O_2_ cultivation conditions. Data represent the mean values of triplicates ±1 SE **E)** Correlation plot of gravimetric and fluorometric Nile-Red lipid content analysis (r^2^ = 0.82).

We next wished to visually observe the effect of nitrogen depletion and H_2_O_2_ treatment on lipid accumulation. After cultivation, we collected and stained cells with Nile red and observed with fluorescence microscopy. We used three different conditions: 1) control, 2) 0.05 mM nitrogen and 3) 4 mM H_2_O_2_. Microscopy results were in strong agreement with our previous results, as lipid accumulation (increased size and number of cytoplasmic lipid droplets) after nitrogen depletion or H_2_O_2_ treatment was higher than the control (Figure 8A–8B–8C).

Moreover, in order to validate the lipid increasing effect of nitrogen limitation and H_2_O_2_ induced oxidative stress, solvent extraction gravimetric lipid analysis in nitrogen depleted (0.05 mM) and H_2_O_2_ containing media (4 mM) were done. We found that, nitrogen limitation led to increased lipid accumulation up to 35%, and H_2_O_2_ induced oxidative stress led to increased lipid accumulation up to 44% as shown in Figure 8D. We also measured the lipid content by Nile red fluorescence analysis for each of these conditions. Consistent with previous studies, we found that gravimetric and fluorometric measurements were correlated (r^2^ = 0.82) (Figure 8E) [17]. These data suggest that exogenously induced oxidative stress triggered by the application of H_2_O_2_ resulted to increased lipid accumulation. Therefore, as previously shown by the fluorometric analyses above, oxidative stress and increased lipid accumulation association is coupled under nitrogen limitation conditions. Induction of lipid accumulation by applying exogeneous oxidative stress inducers such as H_2_O_2_ may assist more effective lipid production compared with biomass production lowering nitrogen starvation strategy.

ROS production resulting from various stress factors is known to affect nearly all cellular processes by impairing the structural stability of functional macromolecules including DNA, proteins and structural lipids. Since the green algae life-cycle is reliant on its photosynthetic activity and cellular integrity, it is crucial to protect against oxidative stress. Otherwise cells can not tolerate oxidative damage and eventually die [47]–[49]. Even though there is limited explanation about the correlation between ROS production and lipid accumulation in algae cultivated under nitrogen depleted conditions. Our results indicate that nitrogen depletion results in ROS accumulation and lipid peroxidation especially at stationary growth phase. This relationship might also be related to survival response of the alga against excessive oxidative stress conditions [7].

Although a few recent studies have suggested an association of ROS levels and cellular lipid accumulation [7], [8], underlying mechanistic principles are not clear. ROS is well demonstrated to modify cellular responses against different stressors in corresponding signal transduction pathways. ROS levels increase in microalgae cells when exposed to the different environmental stresses [9]. In a recent study, nitrogen depletion was shown to correlate with increased ROS accumulation and increased cellular lipid accumulation in a freshwater algae. This study showed that increased MDA concentration is an indicator of membrane peroxidation which implied increased ROS levels. It was suggested that H_2_O_2_ induced exogenous oxidative stress was an effective factor for neutral lipid induction in *C. sorokiniana C3* [50].

Microalgae can modify its photosynthetic system under stress, resulting in a decrease in the gene expression of various proteins forming up the photosystem complexes I and II [51]. Such adjustments are thought to occur for minimizing oxidative stress via decreasing photosynthesis rate [52]. Nitrogen deprivation is closely associated with the degradation of ribulose-1,5-bisphosphate carboxylase oxygenase to recycle nitrogen [53]. Degradation of this protein may result in alterations in photosynthesis rate, which is consistent with the observed decrease in chlorophyll content under nitrogen depleted condition [54]. As a result of decreased photosynthesis rate, overall anabolic reaction flux is severely constrained. In this context, algae cells may favor storage of energetic molecules, such as lipids, instead of consumption.

### Effects of H_2_O_2_ Induced Oxidative Stress on Lipid Accumulation in *Dunaliella tertiolecta* and *Chlamydomonas reinhardtii*

**Figure 9:**
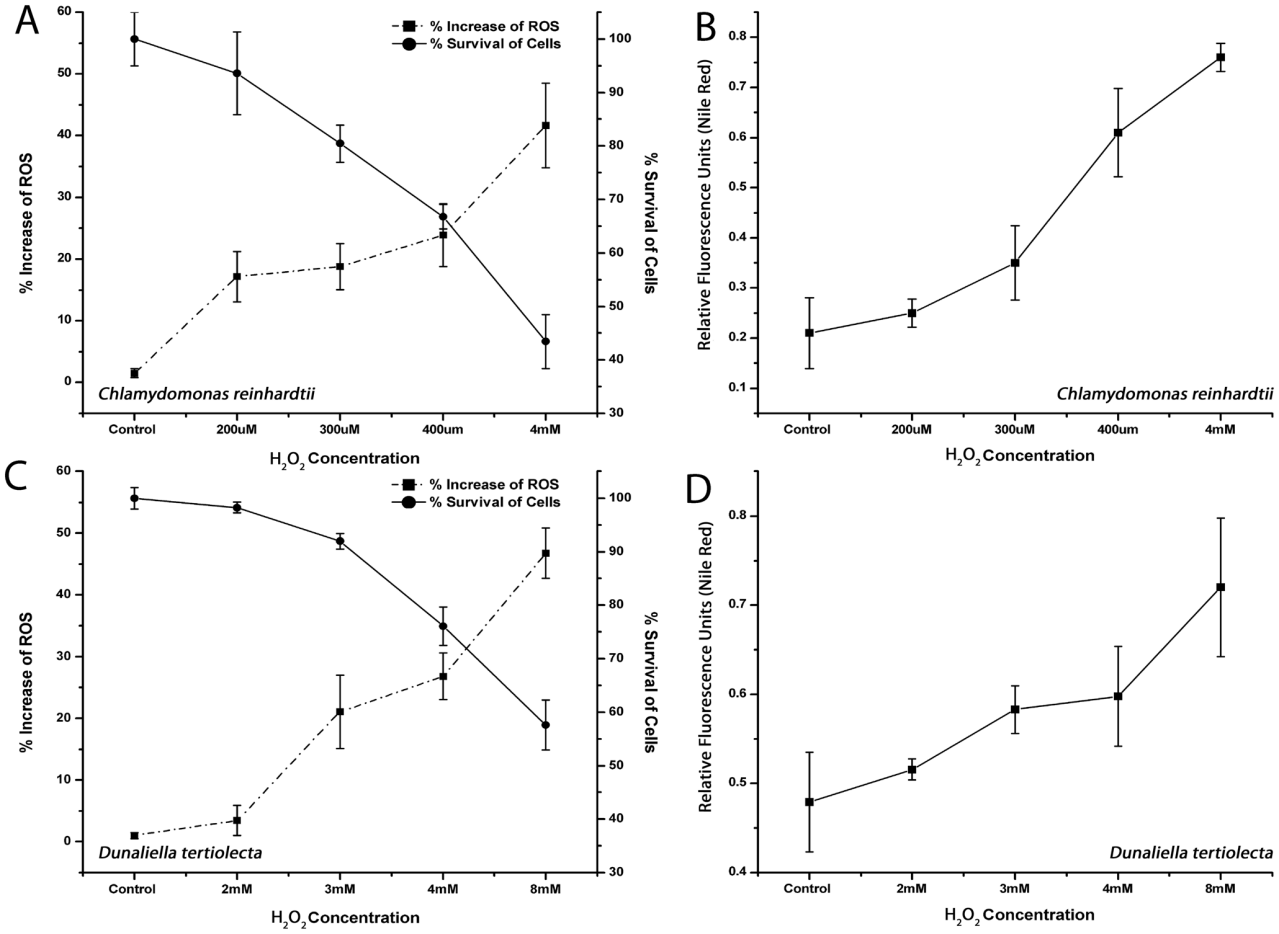
Effect of H_2_O_2_ induced oxidative stress on cellular lipid accumulation of different algae species. **A)** Fluorometric DCFH-DA and FDA analyses showing percantage change of ROS production and percentage change of cell survivability of *Chlamydomonas reinhardtii* cells cultivated under different H_2_O_2_ concentrations. **B)** Fluorometric Nile red analysis showing the effect of different H_2_O_2_ concentrations on cellular lipid accumulation of *Chlamydomonas reinhardtii* cells **C)** Fluorometric DCFH-DA and FDA analyses showing percantage change of ROS production and percentage change of cell survivability of *Dunaliella tertiolecta* cells cultivated under different H_2_O_2_ concentrations. **D)** Fluorometric Nile Red analysis showing the effect of different H_2_O_2_ concentrations on cellular lipid accumulation of *Dunaliella tertiolecta* cells. Data represent the mean values of triplicates ±1 SE.

**Figure 10:**
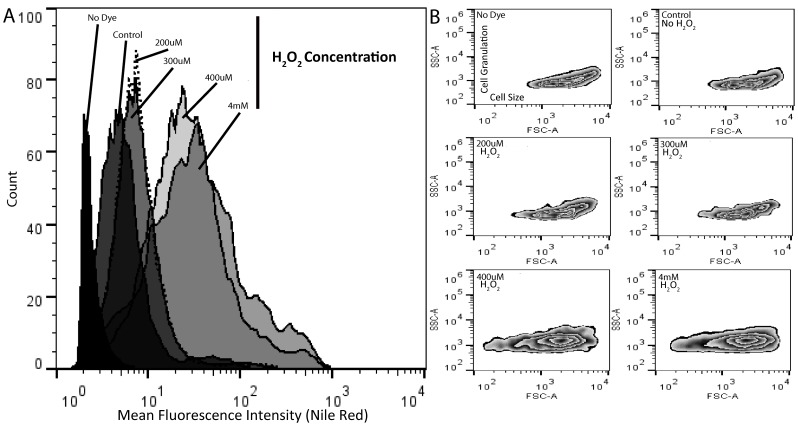
Flow cytometric Nile red, cellular granulation and biovolume analysis of *Chlamydomonas reinhardtii* cells cultivated under different H_2_O_2_ concentrations. **A)** A representative histogram of flow-cytometric analysis of lipid contents under different H_2_O_2_ concentrations. **B)** Zebra-plot of SSC (Side Scatter) and FSC (Forward Scatter) expressing cellular granulation and cellular size, respectively, under different H_2_O_2_ concentrations. Data represent the mean values of triplicates ±1 SE.

Green algae, *Dunaliella tertiolecta* and *Chlamydomonas reinhardtii* were chosen for further investigation and confirmation of the H_2_O_2_ induced oxidative stress effects on cellular lipid accumulation. We cultivated cells in high nitrogen containing media (5 mM) with different concentrations of H_2_O_2_ as described for *Dunaliella salina strain Tuz_KS_01.* We measured ROS by using the DCFH-DA method, cell viability by using the FDA fluorescent assay, and lipid content by using the fluorometric Nile Red method, as described above. *Dunaliella tertiolecta* cells were found to be more tolerant to H_2_O_2_; therefore higher concentrations of H_2_O_2_ were used to demonstrate the cellular response. *Chlamydomonas reinhardtii* cells were treated within the range from 200 μM to 4 mM H_2_O_2_ concentrations while *Dunaliella tertiolecta* cells were treated within the range of 2 mM to 8 mM H_2_O_2_ concentrations. As shown in Figure 9, we found that in response to H_2_O_2_ in both species ROS accumulation and lipid contents increased, while cell survival dramatically decreased at high H_2_O_2_ concentrations. In addition, by using flow cytometric analysis, we observed that lipid increase in response to oxidative stress was accompanied with increased granulation and biovolume in *Chlamydomonas* cells (Figure 10). Neither *Dunaliella tertiolecta* nor *Dunaliella salina strain Tuz_KS_01* cells showed biovolume increase upon treatment of H_2_O_2_ at any experimented concentration.

## Conclusions

Nitrogen depletion has been shown to be an effective inducer of lipid production of various unicellular green algae by previous studies. But few studies have examined the physiological and biochemical mechanisms underlying this response. In this study, we isolated a new halophilic green alga species. We presented evidence supporting previous observations that nitrogen depletion causes oxidative stress and lipid accumulation. In addition, we showed that oxidative stress by itself can cause lipid accumulation, suggesting that lipid accumulation under nitrogen depletion is mediated by oxidative stress. These observations are helpful for utilization of green alga for biodiesel production.

## References

[1] Camacho FG, Rodriguez JG, Miron AS, Garcia MC, Belarbi EH, et al (2007) Biotechnological significance of toxic marine dinoflagellates. Biotechnol Adv 25: 176–194. 17208406 10.1016/j.biotechadv.2006.11.008

[2] Li Y, Horsman M, Wang B, Wu N, Lan CQ (2008) Effects of nitrogen sources on cell growth and lipid accumulation of green alga Neochloris oleoabundans. Appl Microbiol Biotechnol 81: 629–636. 18795284 10.1007/s00253-008-1681-1

[3] Csavina JL, Stuart BJ, Riefler RG, Vis ML (2011) Growth optimization of algae for biodiesel production. J Appl Microbiol 111: 312–318. 21624020 10.1111/j.1365-2672.2011.05064.x

[4] Spolaore P, Joannis-Cassan C, Duran E, Isambert A (2006) Commercial applications of microalgae. J Biosci Bioeng 101: 87–96. 16569602 10.1263/jbb.101.87

[5] Alscher RG, Donahue JL, Cramer CL (1997) Reactive oxygen species and antioxidants: Relationships in green cells. Physiologia Plantarum 100: 224–233.

[6] Mallick N, Mohn FH (2000) Reactive oxygen species: response of algal cells. Journal of Plant Physiology 157: 183–193.

[7] Liu WH, Huang ZW, Li P, Xia JF, Chen B (2012) Formation of triacylglycerol in Nitzschia closterium f. minutissima under nitrogen limitation and possible physiological and biochemical mechanisms. Journal of Experimental Marine Biology and Ecology 418: 24–29.

[8] Li X, Hu HY, Zhang YP (2011) Growth and lipid accumulation properties of a freshwater microalga Scenedesmus sp. under different cultivation temperature. Bioresour Technol 102: 3098–3102. 21055924 10.1016/j.biortech.2010.10.055

[9] Hong Y, Hu HY, Li FM (2008) Physiological and biochemical effects of allelochemical ethyl 2-methyl acetoacetate (EMA) on cyanobacterium Microcystis aeruginosa. Ecotoxicol Environ Saf 71: 527–534. 18054385 10.1016/j.ecoenv.2007.10.010

[10] Gordillo FL, Goutx M, Figueroa F, Niell FX (1998) Effects of light intensity, CO2 and nitrogen supply on lipid class composition of Dunaliella viridis. Journal of Applied Phycology 10: 135–144.

[11] Olmos J, Paniagua J, Contreras R (2000) Molecular identification of Dunaliella sp utilizing the 18S rDNA gene. Letters in Applied Microbiology 30: 80–84. 10728567 10.1046/j.1472-765x.2000.00672.x

[12] Fredslund J (2006) PHY center dot FI: fast and easy online creation and manipulation of phylogeny color figures. Bmc Bioinformatics 7. 10.1186/1471-2105-7-315 PMC1513607 16792795

[13] Levasseur M, Thompson PA, Harrison PJ (1993) Physiological acclimation of marine-phytoplankton to different nitrogen-sources. Journal of Phycology 29: 587–595.

[14] Greenspan P, Mayer EP, Fowler SD (1985) Nile red: a selective fluorescent stain for intracellular lipid droplets. J Cell Biol 100: 965–973. 3972906 10.1083/jcb.100.3.965 PMC2113505

[15] Govender T, Ramanna L, Rawat I, Bux F (2012) BODIPY staining, an alternative to the Nile Red fluorescence method for the evaluation of intracellular lipids in microalgae. Bioresour Technol 114: 507–511. 22464420 10.1016/j.biortech.2012.03.024

[16] Chen W, Sommerfeld M, Hu Q (2011) Microwave-assisted nile red method for in vivo quantification of neutral lipids in microalgae. Bioresour Technol 102: 135–141. 20638272 10.1016/j.biortech.2010.06.076

[17] Chen W, Zhang C, Song L, Sommerfeld M, Hu Q (2009) A high throughput Nile red method for quantitative measurement of neutral lipids in microalgae. J Microbiol Methods 77: 41–47. 19162091 10.1016/j.mimet.2009.01.001

[18] Cole TA, Fok AK, Ueno MS, Allen RD (1990) Use of nile red as a rapid measure of lipid content in ciliates. Eur J Protistol 25: 361–368. 23196050 10.1016/S0932-4739(11)80129-X

[19] Kimura K, Yamaoka M, Kamisaka Y (2004) Rapid estimation of lipids in oleaginous fungi and yeasts using Nile red fluorescence. J Microbiol Methods 56: 331–338. 14967224 10.1016/j.mimet.2003.10.018

[20] Kalyanaraman B, Darley-Usmar V, Davies KJA, Dennery PA, Forman HJ, et al (2012) Measuring reactive oxygen and nitrogen species with fluorescent probes: challenges and limitations. Free Radical Biology and Medicine 52: 1–6. 22027063 10.1016/j.freeradbiomed.2011.09.030 PMC3911769

[21] Liang ZJ, Ge F, Zeng H, Xu Y, Peng F, et al (2013) Influence of cetyltrimethyl ammonium bromide on nutrient uptake and cell responses of Chlorella vulgaris. Aquatic Toxicology 138: 81–87. 23721850 10.1016/j.aquatox.2013.04.010

[22] Schonswetter P, Tribsch A, Barfuss M, Niklfeld H (2002) Several Pleistocene refugia detected in the high alpine plant Phyteuma globulariifolium sternb & hoppe (Campanulaceae) in the European Alps. Mol Ecol 11: 2637–2647. 12453246 10.1046/j.1365-294x.2002.01651.x

[23] Barbarino E, Lourenco SO (2005) An evaluation of methods for extraction and quantification of protein from marine macro- and microalgae. Journal of Applied Phycology 17: 447–460.

[24] Bradford MM (1976) A rapid and sensitive method for the quantitation of microgram quantities of protein utilizing the principle of protein-dye binding. Anal Biochem 72: 248–254. 942051 10.1016/0003-2697(76)90527-3

[25] Wellburn AR (1994) The spectral determination of chlorophyll-a and chlorophhyll-b, as well as total carotenoids, using various solvents with spectrophotometers of different resolution. Journal of Plant Physiology 144: 307–313.

[26] Sabatini SE, Juarez AB, Eppis MR, Bianchi L, Luquet CM, et al (2009) Oxidative stress and antioxidant defenses in two green microalgae exposed to copper. Ecotoxicology and Environmental Safety 72: 1200–1206. 19223073 10.1016/j.ecoenv.2009.01.003

[27] Dhindsa RS, Plumbdhindsa P, Thorpe TA (1981) Leaf senescence - correlated with increased levels of membrane-permeability and lipid-peroxidation, and decreased levels of superoxide-dismutase and catalase. Journal of Experimental Botany 32: 93–101.

[28] Aebi H (1984) Catalase invitro. Methods in Enzymology 105: 121–126. 6727660 10.1016/s0076-6879(84)05016-3

[29] Nakano Y, Asada K (1981) Hydrogen-peroxide is scavenged by ascorbate-specific peroxidase in spinach-chloroplasts. Plant and Cell Physiology 22: 867–880.

[30] Tang HY, Abunasser N, Garcia MED, Chen M, Ng KYS, et al (2011) Potential of microalgae oil from Dunaliella tertiolecta as a feedstock for biodiesel. Applied Energy 88: 3324–3330.

[31] Ho SH, Chen WM, Chang JS (2010) Scenedesmus obliquus CNW-N as a potential candidate for CO2 mitigation and biodiesel production. Bioresource Technology 101: 8725–8730. 20630743 10.1016/j.biortech.2010.06.112

[32] Griffiths MJ, van Hille RP, Harrison STL (2012) Lipid productivity, settling potential and fatty acid profile of 11 microalgal species grown under nitrogen replete and limited conditions. Journal of Applied Phycology 24: 989–1001.

[33] Chen M, Tang HY, Ma HZ, Holland TC, Ng KYS, et al (2011) Effect of nutrients on growth and lipid accumulation in the green algae Dunaliella tertiolecta. Bioresource Technology 102: 1649–1655. 20947341 10.1016/j.biortech.2010.09.062

[34] Sharma KK, Schuhmann H, Schenk PM (2012) High Lipid Induction in Microalgae for Biodiesel Production. Energies 5: 1532–1553.

[35] Lee SJ, Yoon BD, Oh HM (1998) Rapid method for the determination of lipid from the green alga Botryococcus braunii. Biotechnology Techniques 12: 553–556.

[36] Lin QA, Lin JD (2011) Effects of nitrogen source and concentration on biomass and oil production of a Scenedesmus rubescens like microalga. Bioresource Technology 102: 1615–1621. 20875734 10.1016/j.biortech.2010.09.008

[37] Bhaduri AM, Fulekar MH (2012) Antioxidant enzyme responses of plants to heavy metal stress. Reviews in Environmental Science and Bio-Technology 11: 55–69.

[38] Alscher RG, Erturk N, Heath LS (2002) Role of superoxide dismutases (SODs) in controlling oxidative stress in plants. Journal of Experimental Botany 53: 1331–1341. 11997379

[39] del Rio LA, Sandalio LM, Corpas FJ, Palma JM, Barroso JB (2006) Reactive oxygen species and reactive nitrogen species in peroxisomes. Production, scavenging, and role in cell signaling. Plant Physiology 141: 330–335. 16760483 10.1104/pp.106.078204 PMC1475433

[40] Miller SR, Martin M, Touchton J, Castenholz RW (2002) Effects of nitrogen availability on pigmentation and carbon assimilation in the cyanobacterium Synechococcus sp strain SH-94-5. Archives of Microbiology 177: 392–400. 11976748 10.1007/s00203-002-0404-8

[41] Singh SP, Hader DP, Sinha RP (2010) Cyanobacteria and ultraviolet radiation (UVR) stress: Mitigation strategies. Ageing Research Reviews 9: 79–90. 19524071 10.1016/j.arr.2009.05.004

[42] Courchesne NMD, Parisien A, Wang B, Lan CQ (2009) Enhancement of lipid production using biochemical, genetic and transcription factor engineering approaches. Journal of Biotechnology 141: 31–41. 19428728 10.1016/j.jbiotec.2009.02.018

[43] Msanne J, Xu D, Konda AR, Casas-Mollano JA, Awada T, et al (2012) Metabolic and gene expression changes triggered by nitrogen deprivation in the photoautotrophically grown microalgae Chlamydornonas reinhardtii and Coccomyxa sp C-169. Phytochemistry 75: 50–59. 22226037 10.1016/j.phytochem.2011.12.007

[44] Tsukagoshi H (2012) Defective root growth triggered by oxidative stress is controlled through the expression of cell cycle-related genes. Plant Science 197: 30–39. 23116669 10.1016/j.plantsci.2012.08.011

[45] Cash TP, Pan Y, Simon MC (2007) Reactive oxygen species and cellular oxygen sensing. Free Radical Biology and Medicine 43: 1219–1225. 17893032 10.1016/j.freeradbiomed.2007.07.001 PMC2696222

[46] Converti A, Casazza AA, Ortiz EY, Perego P, Del Borghi M (2009) Effect of temperature and nitrogen concentration on the growth and lipid content of Nannochloropsis oculata and Chlorella vulgaris for biodiesel production. Chemical Engineering and Processing 48: 1146–1151.

[47] Hu Q, Sommerfeld M, Jarvis E, Ghirardi M, Posewitz M, et al (2008) Microalgal triacylglycerols as feedstocks for biofuel production: perspectives and advances. Plant Journal 54: 621–639. 18476868 10.1111/j.1365-313X.2008.03492.x

[48] Kobayashi M (2003) Astaxanthin biosynthesis enhanced by reactive oxygen species in the green alga Haematococcus pluvialis. Biotechnology and Bioprocess Engineering 8: 322–330.

[49] Pinto E, Sigaud-Kutner TCS, Leitao MAS, Okamoto OK, Morse D, et al (2003) Heavy metal-induced oxidative stress in algae. Journal of Phycology 39: 1008–1018.

[50] Zhang YM, Chen H, He CL, Wang Q (2013) Nitrogen starvation induced oxidative stress in an oil-producing green alga Chlorella sorokiniana C3. PLoS One 8: e69225. 23874918 10.1371/journal.pone.0069225 PMC3712941

[51] Zhang ZD, Shrager J, Jain M, Chang CW, Vallon O, et al (2004) Insights into the survival of Chlamydomonas reinhardtii during sulfur starvation based on microarray analysis of gene expression. Eukaryotic Cell 3: 1331–1348. 15470261 10.1128/EC.3.5.1331-1348.2004 PMC522608

[52] Nishiyama Y, Yamamoto H, Allakhverdiev SI, Inaba M, Yokota A, et al (2001) Oxidative stress inhibits the repair of photodamage to the photosynthetic machinery. EMBO J 20: 5587–5594. 11598002 10.1093/emboj/20.20.5587 PMC125664

[53] Garcia-Ferris C, Moreno J (1993) Redox regulation of enzymatic activity and proteolytic susceptibility of ribulose-1,5-bisphosphate carboxylase/oxygenase fromEuglena gracilis. Photosynth Res 35: 55–66. 24318620 10.1007/BF02185411

[54] Cakmak T, Angun P, Ozkan AD, Cakmak Z, Olmez TT, et al (2012) Nitrogen and sulfur deprivation differentiate lipid accumulation targets of Chlamydomonas reinhardtii. Bioengineered 3: 343–346. 22892589 10.4161/bioe.21427 PMC3489711

